# Mucus Distribution Model in a Lung with Cystic Fibrosis

**DOI:** 10.1155/2012/970809

**Published:** 2012-10-17

**Authors:** Sara Zarei, Ali Mirtar, Forest Rohwer, Douglas J. Conrad, Rebecca J. Theilmann, Peter Salamon

**Affiliations:** ^1^Computational Science Research Center, San Diego State University, San Diego, CA 92182, USA; ^2^Electrical and Computer Engineering Department, University of California, San Diego, La Jolla, CA 92093, USA; ^3^Department of Biology, San Diego State University, San Diego, CA 92182, USA; ^4^School of Medicine, University of California, San Diego, La Jolla, CA 92093, USA; ^5^Department of Radiology, University of California, San Diego, La Jolla, CA 92093, USA; ^6^Department of Mathematics and Statistics, San Diego State University, San Diego, CA 92182, USA

## Abstract

Cystic fibrosis (CF) is the most common autosomal recessive disease in Caucasians with a reported incidence of 1 in every 3200 live births. Most strikingly, CF is associated with early mortality. Host in flammatory responses result in airway mucus plugging, airway wall edema, and eventual destruction of airway wall support structure. Despite aggressive treatment, the median age of survival is approximately 38 years. This work is the first attempt to parameterize the distributions of mucus in a CF lung as a function of time. By default, the model makes arbitrary choices at each stage of the construction process, whereby the simplest choice is made. The model is sophisticated enough to fit the average CF patients' spirometric data over time and to identify several interesting parameters: probability of colonization, mucus volume growth rate, and scarring rate. Extensions of the model appropriate for describing the dynamics of single patient MRI data are also discussed.

## 1. Introduction

Cystic fibrosis is caused by mutations in the cystic fibrosis transmembrane regulator (CFTR) ion channel gene. The defective gene results in abnormally thick, sticky mucus that affects the lungs, the digestive system, and the circulatory system. CF patients eventually face severe breathing problems, inadequate digestion, and malabsorption of nutrients. They experience intermittent pulmonary exacerbations characterized by dyspnea, cough, sputum production, and sinusitis as a result of a buildup of mucus plugs and microbial biofilms [1]. Over time, these airway infections will cause airway scarring, remodeling, and ultimately respiratory failure.

Our hypothesis is that this scarring, and ultimate remodeling, is primarily due to the contact between the lung lining and the mucus which contains inflammatory cytokines known to induce scars in lung tissue cultures. While the presence of virulent microbes in this mucus is also sure to play a role, such role is again mediated by contact between mucous biofilm and lung tissue. Many of the choices (assumptions) in the model below are predicated on the hypothesis that the distribution of mucus is the key observational variable for a mathematical description of the state of a CF patient.

Mathematical modeling has proved to be useful in the study of chronic diseases such as hepatitis B, lupus, kidney nephritis, mitral regurgitation, and cancer. There, have been quantitative simulations of these diseases based on experimentally validated mathematical models. These models provide an opportunity for the researcher, and eventually the clinician, to address data and information in the context of well-formulated questions and what-if scenarios [2–5].

**Figure 1:**
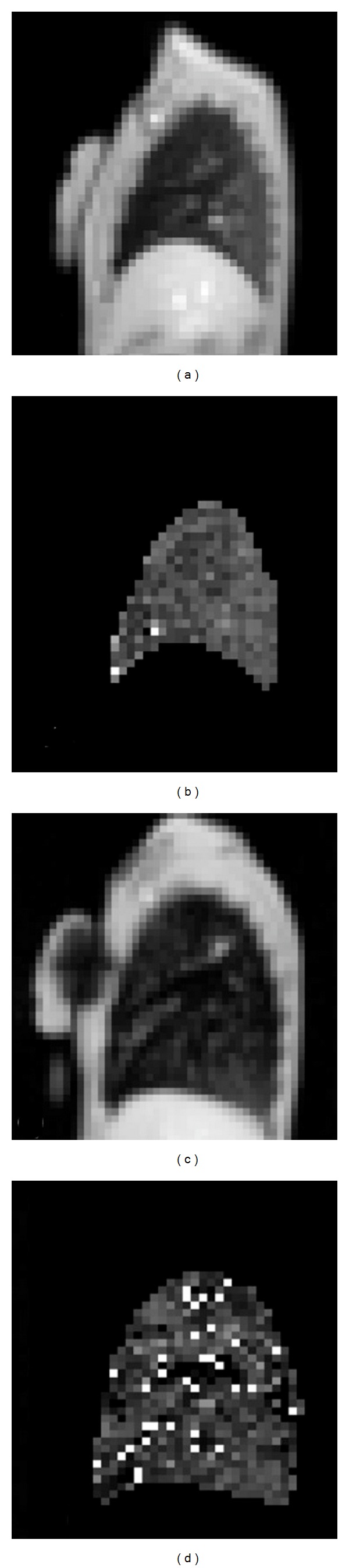
Image data (a, c) and corresponding lung density maps (b, d) for a healthy male (41 yo, FEV1[pred] = 106% (a, b)) and a CF male with severe disease (42 yo, FEV1[pred] = 37% (c, d)) using an MRI methodology as described in a study conducted by Theilmann et al. [6]. The artifact in the chest wall in (d) is due to a metal ring in an installed port-a-cath. The hyperintense regions in the lung density map of the CF subject (d) indicate lung regions full of mucus and regions showing an absence of water are associated with tissue scarring. Neither observation is seen in the healthy subject.

There are several recently developed tools for observing the dynamic distribution and composition of mucus in a CF lung. Predominant among these tools is the ability to image this mucus using MRI rather than the heretofore available computed tomography (CT) scans. The use of the MRI technique makes frequent monitoring of the mucus distribution possible and calls for a theoretical framework with which to make sense of the dynamics of the mucus distribution. Shown in Figure 1 are representative MRI images (a, c) and corresponding lung water density maps (b, d) in the right lung for a healthy age-matched volunteer (a, b), and a CF subject with severe disease (c, d) [6].

The figure shows that mucus is not uniformly distributed in a CF lung. A model predicting the location and growth of infection pockets would be a useful clinical tool.

The model presented below was constructed toward this purpose. The simplest version of the model, described in Section 2, was tested on the average data of an afflicted population. This was achieved by fitting the average course of the disease with constant values of mucus volume growth and scarring rate. The average course of the disease was represented for this work by the mean FVC (FVC, forced vital capacity, is a spirometric test that measures volume of air that can forcibly be blown out after full inspiration maneuver.) values as a function of age for patients in the University of California San Diego Adult Cystic Fibrosis Center (UCSD-ACFC). CF is a chronic disease and FVC is the standard end point for clinical trials. We were able to accurately match our model to the observed data and extract mean parameters of interest: probability of colonization, mucus volume growth rate, scarring rate, and threshold for the progression of the disease.

Our preliminary model is based on the symmetric binary tree structure of an adult lung [7] and assumes constant values for the rates of mucus accretion and scarring in an infected bronchiole. The model is highly modular following the description of our results of the UCSD-ACFC example by describing an implementation for individual patients. The various submodels required here will soon be informed by data characterizing the microbial communities present. Such data comes from metagenomic analyses of sputum samples and gas chromatography/mass spectrometry (gc/ms) analyses of exhaled air. We leave modeling the dynamics of the communities for a different effort and here content ourselves with discussing the net effect on the submodels presented here. 

## 2. Model Assumptions

Our model follows the physiological state of the lung throughout a CF patient's life. The model is modular and consists of submodels individually implemented in the simplest possible way.

**Figure 2:**
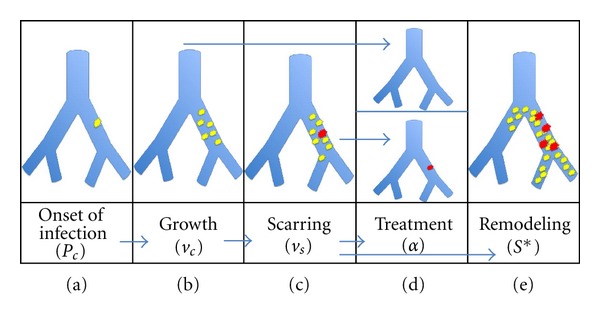
Submodels for the growth and distribution of mucus in a CF lung. Current model is dealing with five aspects of the growth pattern and distribution.

The model assumes the lung airways to be binary branching trees [7–10] extending over 23 generations from the bronchus down to the alveoli using Weibel's measurements of a typical adult lung. To describe the distribution of mucus in the airways requires the submodels shown in Figure 2 and discussed in the following subsections.

### 2.1. Onset of Infection

The onset of infection depends on many factors including season and patient immune state. For the median response in this simplest model, the onset of infection in an airway is assumed to occur with a uniform probability density (*P*_*c*_) per unit area (*A*_*i*_) per unit time over all bronchiole surfaces (Figure 2(a)). Cross-infection is neglected, that is, higher probability of a second infection forming once one has taken root is not considered except for scarred airways which are assumed to be reinfected instantaneously. In addition, overflow from a nearby bronchiole also creates an infected bronchiole with certainty.

### 2.2. Growth of Mucus/Biofilm

This submodel contains the central parameter of the model: the growth rate of mucus volume, *v*_*c*_, velocity of a colony growth per unit time (Figure 2(b)). In reality, *v*_*c*_ surely depends on many factors such as bacterial community composition and strength of immune response. In this simplest model, we have taken this growth rate to be constant, resulting in linear growth. There are certainly other possibilities for simple models, for example, exponential. In fact, linear growth in each bronchiole gives an approximately exponential effect since this growth is proportional to the number of infected bronchioles.

Each infected bronchiole experiences a constant increase in its mucus volume until it becomes full. Once full, the bronchiole infects its mother and daughter bronchioles and all airways distal to the filled bronchiole are assumed to be reversibly nonfunctional.

### 2.3. Scarring

Our scarring submodel is predicated on the assumption that the dominant source of damage to the lung tissue occurs from prolonged contact with mucus filled with highly inflammatory mediators. Thus, our submodel assumes scarring begins in any airway which has had mucus continuously present for more than a certain threshold time. Scarring will accumulate with a constant rate of *v*_*s*_ (Figure 2(c)). Once scarring begins, infection is never completely removed.

### 2.4. Treatment

Treatment is initiated when the patient comes to the clinic in response to an exacerbation (Figure 2(d)). While general agreement as to what constitutes an exacerbation is lacking in this model, we assume that an exacerbation means a certain fraction of operational airway volume is lost due to mucus buildup, given the last previously achieved maximum. Treatment resets all mucus volume to zero. Damaged airways remain infected, while undamaged airways do not. For simplicity, we assume that treatment is triggered when *V*_*L*_ ⩽  *αV*_L_*, where *V*_*L*_ equals current functional airway volume, *V*_L_* equals functional airway volume after last treatment, and *α* equals threshold fraction of operational airways volume.

### 2.5. Remodeling

When damage (scarring) in a bronchiole reaches a certain threshold value (S*≝1), tissue remodeling is initiated making the bronchiole and all airways distal to it irreversibly nonfunctional (Figure 2(e)). This threshold value is taken equal to 1, thereby establishing a unit for scarring.

### 2.6. Community Dynamics, FVC Approximation

 The bacterial composition of infected pockets is likely to have a major effect on the course of the disease in an individual, but for the average response simulated here, we take the composition to be fixed and unknown (new methods such as metagenomics and gas chromatography/mass spectrometry can reveal much about the community types and corresponding disease states of individual patients, see Section 6).

The easiest and most frequent measurements appraising the state of a CF patient are his FVC and FEV1 values (FEV1: forced expiratory volume in one second). Predicting FEV1 values from the distribution of mucus is a difficult fluid mechanics problem requiring the consideration of mixed laminar and turbulent flows. For the present model, we assumed that the percent of functional airway volume represents the percent of normal FVC. When a bronchiole gets completely filled with mucus, air cannot reach the corresponding alveolus (air sac) at the end of that subtree. This results in FVC reduction. In our simulation, we track completely plugged bronchioles and find the total number of inaccessible alveoli. To a good approximation, FVC is just the total volume of the accessible alveoli. In the rest of this paper we will use the number of accessible alveoli as a proxy for FVC.

## 3. The Simulation

**Figure 3:**
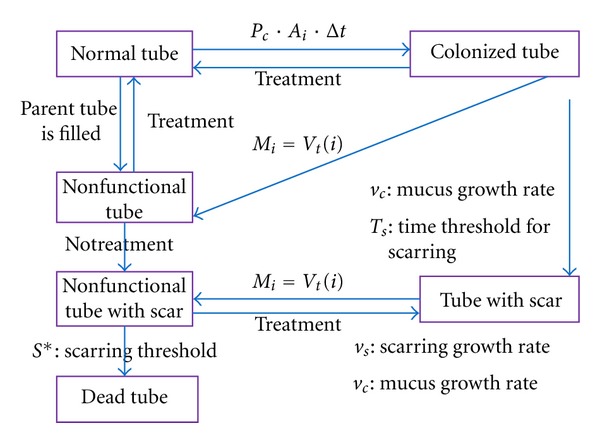
Flowchart of the progression of the disease simulation.

In this model, we simulate the creation, growth, propagation and clearing of mucus in lung airways. The reaction of each individual bronchiole to the colonization in a CF lung, as well as the effect of this colonization on other bronchioles positioned nearby it, has been simulated in this model. The time step was taken as one month and the simulation was run for 600 steps corresponding to 50 years. The simulation code was developed in Matlab. Each bronchiole has a probability *P*_*c*_ per unit time of becoming colonized with a sufficiently large volume of mucus that normal airway function cannot clear. Once colonized, the mucus volume in a bronchiole grows at a constant rate (*v*_*c*_). If the volume of purulence in a bronchiole reaches the volume of a bronchiole, the bronchiole is deemed nonfunctional. Another parameter in the model is scarring which represents a second constant whose value is again dependent on the microbial community. If mucus remains in an airway for a period of time *T*_*s*_, the airway starts scarring at a constant rate (*v*_*s*_). Once scarring inside a airway reaches a threshold (S*), the airway is considered dead and beyond recovery. Therefore, we have two processes that are taking place in each bronchiole: mucus accretion and scarring. Unlike the mucus buildup which responds positively to treatment, scarring does not respond to any kind of treatment. Progression of the disease is displayed as a flowchart in Figure 3, showing the names and meanings of the model parameters.

Due to the binary tree structure of lung airways, each bronchiole has two structural roles. It is the parent airway of its descendant airways and it is a child of its ancestor airways. Once a parent airway becomes nonfunctional, air cannot pass through it. Therefore, its descendant airways are not available for gas exchange. Our simulation follows the behavior of each individual airway during propagation of the infection.

## 4. The Equations

The following equations describe the mathematical model used in our simulation. Formally the equations are time-delay differential equations with switching at certain values of key variables such as mucus volume and scarring.

Each infected site *i* is mapped to a location in the lung and has an associated volume *V*_*i*_ of the airway and a start of infection time *t*_*i*_. For infected site *i*, there are two dynamic parameters: *M*_*i*_: mucus volume and *S*_*i*_: extent of scarring. These in turn determine the values of three logical variables:

*F*_*i*_: filled or not filled. *F*_*i*_ = 1, means that airway (*i*) became reversibly nonfunctional due to the mucus volume reaching the volume of airway. It can be functional again after treatment.

*O*_*i*_: shut or open. *O*_*i*_ = 1 means airway (*i*) became reversibly nonfunctional due to parent airways being plugged. It can be functional again after treatment.

**Figure 4:**
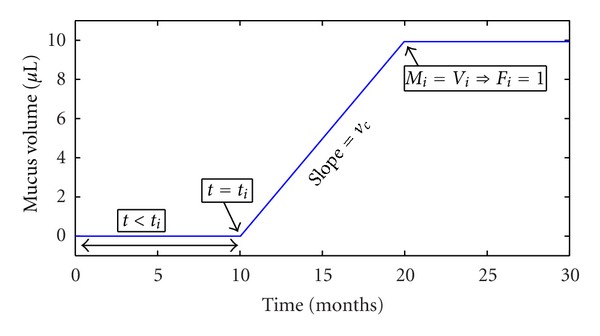
Mucus volume in the 10th generation as a function of time measured from an arbitrary time *t* = 0. For the airway shown, the time of infection is *t*_*i*_ = 10 months when mucus volume starts increasing at rate *v*_*c*_ up to the point where the airway becomes completely filled with biofilm.

**Figure 5:**
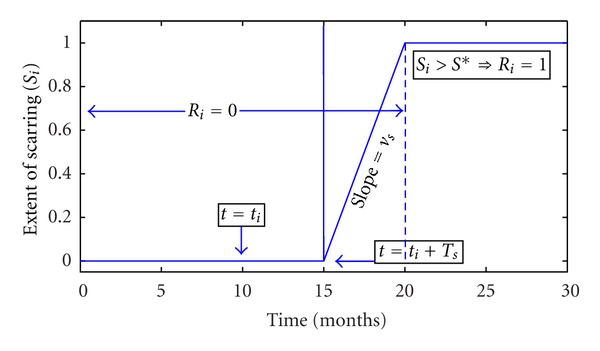
Amount of scarring in the *i*th airway as a function of time measured from an arbitrary time *t* = 0. For the airway shown, the time of infection is *t*_*i*_ = 10 months and scarring begins *T*_*s*_ = 5 months after the infection time. Therefore, after *t* = 15 months airway (*i*) starts getting scars. Scarring grows at rate of *v*_*s*_ until airway (*i*) becomes irreversibly nonfunctional. The *y*-axis shows the extent of scarring (arbitrary units).

*R*_*i*_: remodeled or not remodeled. *R*_*i*_ = 1 means airway (*i*) became irreversibly nonfunctional by reaching the scarring threshold. No treatment can help an airway at this stage. For site index *i* = 1 to *n*, we have

(1) $$\begin{array}{c} F_{i}=H\left(M_{i}-V_{i}\right),\\ O_{i}=H\left(\sum_{j=1}^{n}F_{\text{ancestor}\;(i)}-1\right),\\ R_{i}=H\left(S_{i}-S^{*}\right),\\ \frac{dM_{i}}{dt}=\left(1-F_{i}\right)\cdot H\left(t-t_{i}\right)\cdot v_{c},\\ \frac{dS_{i}}{dt}=\left(1-R_{i}\right)\cdot H\left(t-\left(t_{i}+T_{s}\right)\right)\cdot v_{s}, \end{array}$$

where *v*_*c*_ and *v*_*s*_ represent the mucus and scarring growth rate, respectively. In the above equations, *H* represents the Heaviside step function whose value is zero for negative arguments and one for positive arguments. Figures 4 and 5 show the mucus volume and amount of scarring in a single airway over time. In addition, the number of infected sites (*n*) at time (*t* + Δ*t*) is the sum of the infected sites at time (*t*) plus the sites that became infected at time (*t* + Δ*t*), that follow a Poisson probability distribution, and the sum of all infected neighbors in the event that the mucus volume reaches the volume of the airway:

(2) n(t+Δt)=n(t)+∑uninfectedairway iPois(λi)+∑j=1n(Fj(t+Δt)−Fj(t))·neighbors (j),

where *λ*_*i*_ = *P*_*c*_ · *A*_*i*_ · Δ*t*, *A*_*i*_ = bronchiole (*i*) surface area.

## 5. Results

Patient data (*n* = 200) was obtained from the UCSD adult CF clinic database: gender, height, age, and FVC. Using Hankinson's spirometric reference equations [11], we calculated the corresponding normal lung volume of each CF patient according to their gender, age, and height. We then normalized each individual FVC value by converting the value to a percent of their corresponding normal lung FVC and calculated the average FVC at each age from 18 to 50. We assume that this number equals the average percent of functional airway volume.

 We used the Nelder-Mead optimization method [12] to find the model parameters which fit the average FVC of the CF patient data from the patient registry. It took 24 iterations of the Nelder-Mead algorithm to converge. The convergence determined unique values of the leading digits for all the parameters. At each iteration, the simulation was run 100 times and the average functional airway volume (3) was collected from age 18 to 50. The total runtime was about eight hours using Dulcinea CPU clusters from Computational Science Research Center (CSRC) at San Diego State University. It contains 12 workstations of Dual-Quad Xeon (R) CPUs which give a total of 96 computational nodes in parallel [13]

(3) $$\begin{array}{c} \bar{\text{FVC}}\left(t\right)=\frac{1}{100}\sum_{i=1}^{100}{\text{FVC}}_{i}\left(t\right), \end{array}$$

(4) $$\begin{array}{c} \text{S}{\text{E}}_{\text{FVC}}\left(t\right)=\sqrt{\frac{1}{100}\sum_{i=1}^{100}{\left({\text{FVC}}_{i}\left(t\right)-\bar{\text{FVC}}\left(t\right)\right)}^{2}}, \end{array}$$

where *t* is age in years from 18 to 50, FVC(*t*) = Forced vital capacity at age (*t*), and SE_FVC_(*t*) is standard estimate of error at age (*t*).

**Figure 6:**
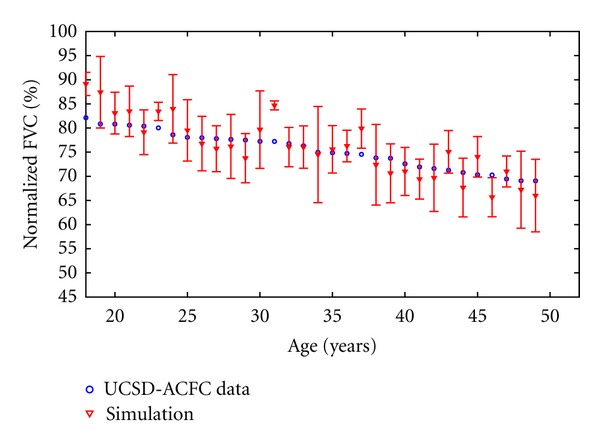
Predictions from the physiological model (lines) versus the CF patient data (circles) from the University of California San Diego Adult Cystic Fibrosis Center. The normalization is based on FVC as a percent of FVC for a healthy lung.

The resulting fit including error bars (4) can be viewed in Figure 6. We found the optimized value of our parameters in a way that our model closely resembles the mean and the standard deviation of real data. Figure 6 shows the predictions of the physiological model versus what is observed on average in the CF patient FVC data. As depicted in Figure 6 based on registry data, on average CF patients start with almost 95 percent of a healthy lung at the age of 18. This value drops down to almost 65 percent by the age of 50.

The corresponding mean squared percent error (MSPE) is 0.95 which indicates the extent to which our model matches the average FVC data from the patient registry

(5) $$\begin{array}{c} \text{PE}\left(t\right)=\frac{\bar{\text{FVC}}\left(t\right)-\text{FV}{\text{C}}^{*}\left(t\right)}{\text{FV}{\text{C}}^{*}\left(t\right)}\cdot 100, \end{array}$$

where PE is percent error and FVC*(*t*) is average FVC at each age from the registry data

(6) $$\begin{array}{c} \text{MSPE}=\frac{\left(\sum_{t=18}^{t=50}\text{PE}{\left(t\right)}^{2}\right)}{n},\;\text{where}\;n=33. \end{array}$$

The optimized parameter values are shown in Table 1. In addition, we conducted a sensitivity analysis by varying one parameter at a time and calculating the corresponding amount of change in total MSPE (6). Each parameter was changed by 1% from its optimized value, while the rest of the parameters were held constant at their optimized value.

Table 1 also shows the variation in total mean square error as the result of 1% change in each parameter. We found that mucus growth rate (*v*_*c*_) is the most sensitive parameter in our model to an extent that 1% variation in its value can almost triple the total mean percent squared error. Subsequently, due to the high sensitivity of the goodness of fit to changes in the mucus growth rate, the optimized value for this parameter converged much faster compared to the rest of the parameters in our model.

The table displays the optimized parameter values and their corresponding sensitivity. The sensitivity was computed by changing the parameter value by 1% and observing the resulting change in MSPE.

The optimized value can be interpreted as follows. Based on the *P*_*c*_ value we obtained, there are almost 70 newly colonized sites every month in a CF lung. Number of newly colonized sites per month = *P*_*c*_ × total surface area of the airways.

**Figure 7:**
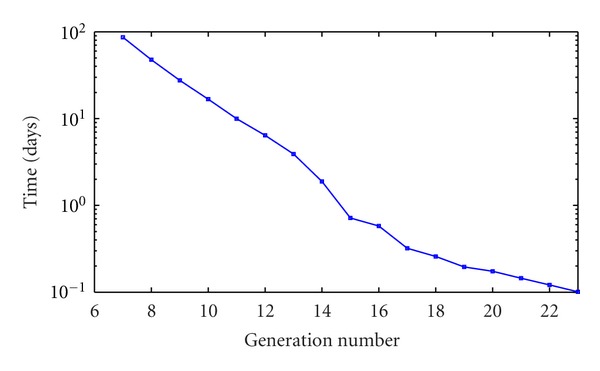
To help with the interpretation of *v*_*c*_, this figure displays the total elapsed time for one infected bronchiole to become completely filled with mucus at each generation.

 According to Figure 7, for the *v*_*c*_ parameter that we fitted, generations 17 and higher would become completely blocked with mucus in less than five hours. Furthermore, the *v*_*s*_ parameter in our model suggests that it will take 7 months to initiate the scarring once a bronchiole has been colonized. Finally, based on the scarring growth rate, it will take almost 3.5 years for a bronchiole that has been colonized to become irreversibly restructured (S*/*v*_*s*_ ≈ 43.5  months ≈ 3.5 years). According to this result, it is important for CF patients to be regularly checked by their physicians in order to prevent initiation of scarring.

## 6. The Next Step

The current model is preliminary in several respects. In particular, it assumes only one type of microbial community, a single probability of infection onset independent of proximity to other pockets of infection present in the lungs, no healing, and so forth. For future models, we will include submodels for community dynamics, immune response, and therapy implemented. Additionally, we will fit the models to individual patient data.

**Figure 8:**
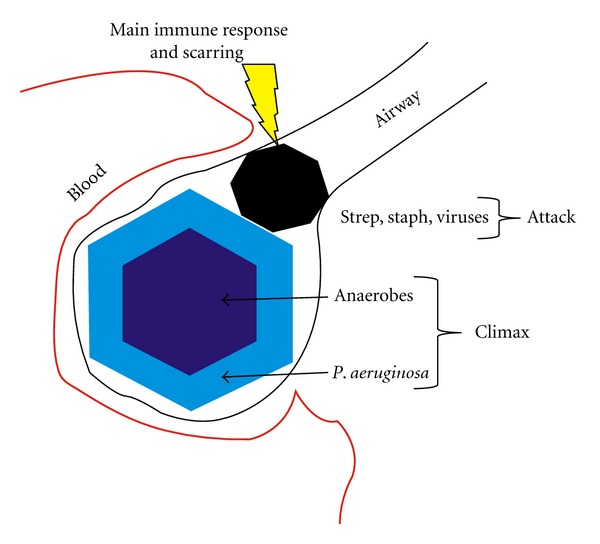
Attack and Climax communities for cystic fibrosis. The Attack communities consist of pathogens like *Streptococcus* spp., *Staphylococcus* spp., and eukaryotic viruses. The Attack communities elicit strong immune responses and scarring. The scar tissue is colonized by the Climax community, which consists of the facultative anaerobe *P. aeruginosa* at the periphery and strict anaerobes in the center. The figure depicts a cross-section through a clogged airway.

The submodels will benefit from the present model with the use of good starting parameter values. It is reasonable to expect that elaborating this model toward realism would still leave the parameters identifiable, especially when FVC, FEV1, and treatment data are supplemented with information gleaned from metagenomics and MRI. From our current understanding of CF, we believe that grouping the microbial communities into two types, Attack and Climax, can give a good representation of the dynamics of mucus distribution in a CF lung (Figure 8). In CF, the mucosal surface is not cleared and is colonized by opportunistic pathogens. Phage diversity increases because the phage prey on these microbes and more eukaryotic (e.g., human) viruses appear to get “stuck” in the mucus. Over the course of a patient's life, a *P. aeruginosa* community becomes entrenched and includes strict anaerobes [14]. We are calling this the Climax community. In contrast, the Attack community changes often and may include *Streptococcus *spp., *Staphylococcus* spp., and eukaryotic viruses (e.g., influenza). In our model, the Attack community creates scarred areas that can be colonized by the Climax community. Treatments may eradicate the Attack communities, but it is unlikely that the Climax communities are ever completely eradicated.

The growth rate for Climax communities is very small whereas for Attack communities it is large. Depending on the values set for these parameters, the two-community type model can display a wealth of behavior that covers the spectrum of observations. Fitting parameters to individual patient records can provide a tool for the clinician to determine the optimal treatment for each individual patient.

Once we collect information about the two types of communities, we can recalculate the equations for each community separately and individualize the parameter values such as growth rates of mucus and scarring. The number of infected sites at time *t* follows a recurrence relation for each site-type *e* ∈ {attack, climax} as follows:

(7) ne(t+Δt)=ne(t)+∑uninfectedairway  iPoise(λi)+∑infected  sitese(Fj,e(t+Δt)−Fj,e(t))·neighbors  (i),

where *λ*_*i*_ = (*P*_*c*_ · *A*_*i*_ · Δ*t*). In addition, for 1 ≤ *i* ≤ *n*_*e*_

(8) $$\begin{array}{c} \frac{dM_{i}}{dt}=\left(1-F_{i,e}\right)\cdot H\left(t-t_{i}\right)\cdot v_{c,e},\\ \frac{dS_{i}}{dt}=\left(1-R_{i,e}\right)\cdot H\left(t-\left(t_{i}+T_{s}\right)\right)\cdot v_{s,e}. \end{array}$$

The physiological portion of the next chapter of the modeling effort will work to identify the above model (two sets of parameter values, one for each type of communities) from FVC and FEV1 data supplemented with metagenomic, MRI, and treatment information. It is expected that various alternative forms of the infection/reinfection probabilities will have to be explored.

## 7. Conclusions

 In conclusion, our model has been used to adjust the probability of onset of infection at a site (per unit area), rate of mucus buildup, scarring rate, and scarring threshold to mimic the UCSD-ACFC data. The parameters revealed clinically useful information such as the time required for bronchioles at different generations to become either reversibly or irreversibly nonfunctional. Furthermore, based on the value of *P*_*c*_, we now have an estimate of the total number of newly colonized sites per month in a CF lung. According to our results, on average generations 15 to 23 can become completely saturated with mucus in less than a day. In addition, it takes almost 3 years for a colonized bronchiole to get remodeled. Therefore, treating CF patients with a specific inhaler that can target these smaller airways may reduce the number of irreversibly restructured bronchioles in their lungs. Using an individual patient's FVC history, our model can estimate mucus growth rate, scarring rate, and threshold value specifically for that patient. This can be used as a reference tool to estimate different treatments' efficacy.

A full model capable of adjusting the parameters according to the microbial community in the lungs and the treatment administered (e.g., timing of antibiotic administration, types of antibiotics, and steroids) would be clinically useful. The present paper should be taken as a proof-of-concept step toward that goal. This will provide an opportunity for the researcher, and eventually the clinician, to access a framework for quantitative predictions.

For the next step, we can use MRI data to improve the model by spatially tracking the presence, growth, and clearing of infections. Once the parameters of the model have been identified, simulations of different treatment scenarios and hypothesized effects can be run and compared to the database, allowing several iterations of the (model/predict/adjust) cycle [15] fitted to individual patient data. Finally, one can imagine many interesting and useful GUI programming implementations of the model that will enable medical doctors to interact with the simulation and tailor their treatment based on contrasts between predicted and observed scenarios. This will provide feedback to improve the model even further.

## Figures and Tables

**Table 1:** Nelder-Mead result and sensitivity analysis.

Parameter	Value	Unit	MSPE variation
*P*_*c*_	6.0 × 10^−6^ ± 0.5 × 10^−6^	mm^−2^/month	8%
*v*_*c*_	11.0 ± 0.5	mm^3^/month	280%
*T*_*s*_	7.0 ± 0.5	Month	15%
*v*_*s*_	0.0230 ± 0.0005	Scars/month	28%
